# The Association of the *COL27A1* rs946053 and *TNC* rs2104772s with Tendinopathies: A Case–Control Study in High-Level Croatian Athletes

**DOI:** 10.3390/genes16080935

**Published:** 2025-08-04

**Authors:** Goran Vrgoč, Saša Janković, Damir Knjaz, Ivana Duvnjak Orešković, Gordan Lauc, Nina Šimunić-Briški

**Affiliations:** 1 Faculty of Kinesiology, University of Zagreb, 10000 Zagreb, Croatia; sjanko@kif.unizg.hr (S.J.); damir.knjaz@kif.unizg.hr (D.K.); 2 Department of Orthopaedic Surgery, University Hospital “Sveti Duh”, 10000 Zagreb, Croatia; 3 Genos Ltd., 10000 Zagreb, Croatia; iduvnjak@genos.hr (I.D.O.); glauc@genos.hr (G.L.); nbriski@genos.hr (N.Š.-B.); 4 Faculty of Pharmacy and Biochemistry, University of Zagreb, 10000 Zagreb, Croatia

**Keywords:** collagen 27 alpha1, tenascin C, soft tissue injury (STI), single-nucleotide polymorphism (SNP), genetic association study, Achilles tendinopathy, sport injury

## Abstract

**Background/Objectives:** The increased risk of developing tendinopathies in athlete populations has led to investigations of several genes associated with tendon properties, suggesting that some individuals have a greater genetic predisposition for developing tendinopathies. The main purpose of this study was to investigate how the functional polymorphisms within the *COL5A1*, *COL27A1* and *TNC* genes impact the risk of developing tendinopathies in high-level Croatian athletes. **Methods**: For this case–control genetic study, we recruited 63 high-level athletes with a diagnosis of tendinopathies and 92 healthy asymptomatic individuals as controls. All individuals were genotyped for three single-nucleotide polymorphisms (SNPs) within the *COL5A1*, *COL27A1* and *TNC* genes using the pyrosequencing method. **Results:** *TNC* rs2104772 TT (*p* = 0.0089) and the T-T-T haplotype (*p* = 0.0234), constructed from rs12722, rs946053 and rs2104772, were significantly overrepresented in cases versus controls, implicating a predisposition for tendinopathies. *COL27A1* rs946053 GG (*p* = 0.0118) and the G-A-C haplotype (*p* = 0.0424), constructed from rs12722, rs946053 and rs2104772, were significantly overrepresented in controls, implicating a protective role. **Conclusions:** These results further support associations between functional polymorphisms within the *COL27A1* and *TNC* genes and the risk of tendinopathies in high-level athletes. Further research is needed to replicate these results in various populations and larger cohorts.

## 1. Introduction

Regular physical activity benefits those who participate, even though it also increases the risk of injury. The sedentary lifestyle that is usually led by recreational athletes prior to returning to their regular sport activity is often an underlying cause of sudden overuse injuries that affect tendons, while tendon ruptures and tendinopathies are serious injuries affecting professional athletes, causing pain and dysfunction and adding to prolonged recovery and return to competing [1,2]. The tendons’ role is to facilitate smooth joint movements, but through that process, tendons are under great strain when transmitting force from muscle to bone. Achilles tendon ruptures are more frequent in males than in females, with ratios ranging from 2:1 to 12:1 [3,4], just as the prevalence of Achilles tendinopathy seems to be increasing through the population, ranging from 10% in the general population to up to 50% among professional athletes [5,6]. Tendinopathies are common overuse injuries associated with sports; they can be in acute or chronic states, mostly affecting the Achilles, patellar, rotator cuff and forearm extensor tendons [7]. Different extrinsic and intrinsic factors contributing to their development have been reported. Extrinsic factors include occupation, sport, physical load, errors in performing exercises and other factors, while intrinsic factors include age, gender, nutrition, anatomical variants, joint laxity and genetic susceptibility [8,9]. Tendons have a highly organized structure, made of tightly packed bundles of fibrils. Collagen is the most abundant protein in tendons, constituting up to 80% of the total dry mass. Glycoproteins, proteoglycans (such as elastin and tenascin C) and other proteins make up the rest of the tendon dry mass. Type I collagen makes up most of the collagen dry mass, while type III and type V collagen and a few others account for the rest [10]. Type V collagen, encoded by the *COL5A1* gene, regulates the diameter of type I collagen fibrils; it consists of triple α1 chains and appears in tissues where type I collagen is expressed [11,12]. The *COL5A1* gene is located on the long arm of chromosome 9 (9q32-q34). Single-nucleotide polymorphisms (SNPs) normally occur throughout the genome, on average in every 100–300 base pairs, and account for up to 90% of the changes within the genome, resulting in roughly 4 to 5 million SNPs. Several SNPs have been found and associated with Achilles tendon injuries, anterior cruciate ligament rupture and a range of motion measurements [13,14,15,16,17]. Just like *COL5A1*, the *TNC* gene is also located on chromosome 9, 19.6 Mbp upstream of *COL5A1*, encoding the tenascin C glycoprotein that is expressed in a highly constrained manner in embryonic tissues, as well as in adult tissues during remodeling and wound healing [18,19]. The extracellular matrixes of musculoskeletal tissues that transmit mechanical forces and are exposed to high levels of stress have high levels of TNC expression [20]. TNC glycoprotein plays an important role in the regulation of cell–matrix interactions. Polymorphisms within the *TNC* gene have been associated with Achilles tendinopathy [21,22], allergic diseases [23] and adult asthma [24]. Also, in the proximity of *TNC* and *COL5A1*, there are some other genes that may be of interest. The *COL27A1* gene encodes the homotrimeric type XXVII fibrillar collagen that provides the structural framework and tensile strength and is highly conserved in vertebrates [25,26]. The risk of Achilles tendinopathy was significantly associated with the haplotype consisting of *TNC* and *COL27A1* sequence variants in the Caucasian population [27]. The aim of this study was to investigate a possible association between the *COL5A1* rs12722, *COL27A1* rs946053 and *TNC* rs2104772 polymorphisms and tendinopathies in Croatian athletes. The hypothesis of this study was that polymorphisms are associated with the incidence of tendinopathy occurrence and that there would be a difference in genotype frequency and haplotypes between affected and unaffected athletes.

## 2. Materials and Methods

### 2.1. Individuals

A total of 155 individuals were recruited between 2016 and 2017 and included in a case–control genetic association study. All individuals were unrelated and physically active athletes from group sports (football, handball, basketball), and a very small percentage (around ~10%) were athletics athletes—medium- and long-distance runners.

The Achilles tendinopathy (TEN) group had clinically diagnosed Achilles tendinopathy, with symptoms including progressive pain, tendon swelling or changes in the lesion thickness and sensitivity to palpation. All confirmed tendinopathies in the TEN group were chronic injuries. Athletes in the TEN group had problems with tendinopathies during their active sports career, and some had to stop with training and competitions a few times during their careers for different medical treatments. Injuries occurred on various occasions but in most cases during periods of physical overload. The TEN group consisted of 63 individuals (47 male and 16 female, average age of 32 years), most being high-level competing athletes in their prospective sports, with intensive training sessions conducted three to seven times a week under the supervision of a professional trainer.

TEN group individuals were chosen in the Orthopedic Clinic by their team medical doctor (orthopedic surgeon). The control group (CON) included 92 individuals (72 male and 20 female, average age 39.0 years) who had completed their active careers and had no tendinopathies during their active careers based on their self-reported history. Both the CON group and TEN group were professional competing athletes. Professional competing athletes in the TEN group have been affected by chronic tendinopathy during their competing career, while for the control group, to ensure that they will not suffer from chronic tendinopathy later in their competing career, we decided to recruit retired athletes who have not had any chronic tendinopathies throughout their professional competing career to maximize the amount of exposure.

This study was approved by the Ethics Committee of the School of Medicine, University of Zagreb, Croatia, and by the Ethics Committee of the Faculty of Kinesiology, University of Zagreb, Croatia. All individuals have signed an informed consent for providing biological samples.

### 2.2. Genotyping

Genomic deoxyribonucleic acid (DNA) was isolated from collected buccal swab samples using a reagent set QIAamp DNA Mini kit (Qiagen, Germantown, MD, USA). Amplification of targeted fragments was performed by polymerase chain reaction (PCR) using specific primer pairs for *COL5A1* rs12722, *COL27A1* rs946053 and *TNC* rs2104772 T>A, listed in Table 1 (Metabion, Planegg/Steinkirchen, Germany), and PyroMark PCR Kit (Qiagen, Germantown, MD, USA).

Amplified fragments were processed by the pyrosequencing method (PyroMark Q24, Qiagen, Germantown, MD, USA) according to the manufacturer’s protocol using specific sequencing primers listed in Table 2 (Metabion, Planegg/Steinkirchen, Germany) and analyzed by PyroMark Q24 software (v 2.0.6, Qiagen, Germantown, MD, USA).

### 2.3. Statistical Analysis

Allelic, genotypic and haplotype differences were analyzed using an odds ratio method, Statcalc program (AcaStat software, Orange County, FL, USA). Differences were considered statistically significant if the *p*-value was *p* < 0.05. For haplotype analysis we used Phase software (Matthew Stephens Laboratory, University of Chicago, IL, USA). The Hardy–Weinberg equilibrium and linkage disequilibrium analyses were performed using the Arlequin software version 3.5 (Genetics and Biometry Laboratory, University of Geneva, Geneva, Switzerland). Based on previously reported studies [13,15,28], we used groups of similar size in this study while investigating genotype effects on various soft-tissue injuries, as those group sizes were proved to be large enough for the detection of the results with established significance. The Bonferroni correction was not used in this study since it is considered to be too conservative [29]. *p*-values were adjusted for false discovery rate (FDR) using the Benjamini–Hochberg procedure for the adjusted *p*-value. It was applied separately for genotypic, allelic and haplotype data.

## 3. Results

Individuals entering this study were divided into two groups: the TEN group with diagnosed tendinopathy and the CON group with no prior tendinopathy injuries during the active competing period of their professional career, based on their self-reported data. It is important to notice that the CON group has significantly higher weight and body mass index (BMI) and is older than the TEN group, which is explained by the changes in lifestyle once professional athletes had retired. The average height of both the TEN and CON group is comparable, and for the purpose of this study it was assumed that weight and BMI of the CON group at the peak point of retired athletes’ careers were comparable to those of the TEN group. Data presented in Table 3 are matched for height, gender and ethnicity but not for BMI and weight as explained earlier.

None of the rs12722 genotypes has significant relevance in our cohort. Genotype frequencies were significantly different between TEN and CON groups. the GG genotype of rs946053 was overrepresented in controls when compared with cases and therefore classified as protective, while the TT genotype of rs2104772 T>A was significantly overrepresented in the TEN group and is associated with a higher risk of tendinopathies. In a similar manner, allelic frequencies were significantly different between two groups. The G allele of rs946053 and the rs2104772 A allele were significantly overrepresented in controls and therefore classified as protective variants, while T alleles of both rs946053 and rs2104772 were significantly overrepresented in cases when compared to controls, so they were considered a predisposition for the development of tendinopathies. T and C allele of rs12722 did not show associations. All polymorphisms conformed to the Hardy–Weinberg equilibrium (HWE) in both cases and the control group (Table 4).

Haplotypes of all possible combinations of SNPs rs12722, rs946053 and rs2104772 T>A were constructed and analyzed. After correcting for FDR, the T-T-T haplotype was considered as predisposition for the development of tendinopathies, while the G-A-C haplotype was considered as protective, as presented in Table 5.

## 4. Discussion

Our understanding of molecular mechanisms underlying soft tissue injuries is still limited. Different candidate gene variants are researched daily. Some of the previously reported variants have been successfully replicated in other populations, while some have stayed significant for one population only. Considering previous studies conducted on other populations, this study investigated further variations within *COL5A1*, *COL27A1* and *TNC* genes and related risks of developing tendinopathies. The main finding of our study conducted in a cohort of Croatian competing athletes suggested that the TT genotype of *TNC* rs2104772 T>A and the T-T-T haplotype constructed from *COL5A1* rs12722, *COL27A1* rs946053 and *TNC* rs2104772 T>A had a significant association with the risk of developing tendinopathies. On the other hand, the GG genotype of *COL27A1* rs946053 suggested the protection of tendinopathies as well as the G-A-C haplotype constructed from *COL5A1* rs12722, *COL27A1* rs946053 and *TNC* rs2104772 T>A.

Type V collagen exists in multiple isoforms, most of which include an alpha chain encoded by the *COL5A1* gene. This gene is located on chromosome 9q34.2–q34.3, a region linked to a higher risk of tendon injuries [30]. Mutations in *COL5A1* are also closely associated with classical Ehlers–Danlos syndrome—a genetic connective tissue disorder marked by joint laxity and fragile tendons and ligaments [31,32]. The 3′-untranslated region (3′UTR) of mRNA plays an important role in regulating gene expression after transcription. Emerging evidence suggests that single-nucleotide polymorphisms (SNPs), such as rs12722 found in the 3′UTR of *COL5A1*, may contribute to various diseases [33]. This specific SNP could alter the 3′UTR’s secondary structure, potentially affecting mRNA stability. As a result, this may impact the formation of collagen fibrils and influence their properties and function [34].

Fibrillar collagens provide the principal structural framework and strength to connective tissues, including tendons, cartilage and skin. Located on chromosome 9q32–33, the *COL27A1* gene spans 156 kilobases and comprises 61 exons [35]. It is responsible for encoding type XXVII collagen, with its predicted protein product consisting of 1860 amino acids [25]. Type XXVII collagen contributes to the tensile strength and structural integrity of tendons, which are essential for smooth joint movement and for withstanding the high mechanical strains encountered during force transmission from muscles to bones [27]. Many previous studies have suggested that genetic factors with the strongest evidence of association involved polymorphisms within *COL5A1*, *COL27A* and *TNC* genes, as well as matrix metaloproteinase-3 (MMP3) and estrogen-related receptor beta (ESRRB) [14,15,16,17,21,27,36,37]. Mutations or variants within COL27A1 can potentially affect tendon quality (strength or flexibility), possibly leading to poorer tendon function or changes in the stability of tendon attachments [27].

The *TNC* gene, situated on the long arm of chromosome 9, spans more than 100 kilobases and contains 29 exons. It is transcribed by a single promoter, which is influenced by regulatory elements—both activating and repressive—located within the first untranslated exon. A total of 1167 SNPs have been identified in the vicinity of the gene, with 67 found within its coding region [38]. In tendons, tenascin-C is highly expressed at sites of high mechanical strain, including myotendinous and osteotendinous junctions, and its upregulation is observed in pathological conditions such as tendinopathy [20]. The SNP rs2104772 in the TNC gene results in a leucine-to-isoleucine substitution within a fibronectin type III domain of the protein. Studies in human cohorts show that this variant is associated with a lower tenascin-C protein content, which may impair its molecular elasticity, affecting the protein’s function in ECM remodeling and cell–matrix interactions [24]. The presence of the T allele (particularly the T/T genotype) has been associated with a higher risk of Achilles tendinopathy and tendon regeneration failure. This may be due to the altered structure and mechanical properties of tenascin-C, leading to compromised tendon adaptation and healing following injury [27].

September et al. investigated the *COL5A1* gene and showed that individuals with the CC genotype of rs12722 were predisposed to Achilles tendon injuries in South African and Australian populations [14]. Following these findings, Brown et al. investigated *COL5A1* rs12722 further in the British cohort, but similarly to our own, in the European cohort, the CC genotype was not significant in AT pathology. Although *COL5A1* rs12722 was not significantly overrepresented in the AT group by itself, three inferred allele combinations constructed from rs12722, rs3196378 and rs71746744 within the *COL5A1* gene were identified as risk modifiers [39]. A study conducted on the population of young academic soccer players connected the CC genotype and C-allele carriers for *COL5A1* rs12722 with predisposition to more soft tissue and ligament injuries, indicating that these associations depend on maturity status due to the phase of physical development of these tissues [40]. Other than age, some other factors, such as gender and ethnicity, should be taken into account when identifying risk connected to genetic variants. Figueiredo et al. showed in their study on rotator cuff tear that the C/T haplotype for *COL5A1* rs3196378 and rs11103544 has a protective effect but only for males [41].

Contrary to these studies, Heffernan et al. observed a large cohort of elite rugby players and the associate C allele of rs12722 and rs3196378 with protective properties against tendon injuries and lower incidence of muscle cramping but also reported a generally greater frequency of allele C in players compared to the control group [42].

Saunders et al. genotyped Australian and South African populations for four polymorphisms within the *COL27A1* gene (rs946053, rs753085, rs1249744 and rs4143245) and three within *TNC* gene (rs2104772, rs1330363 and rs13321), resulting in the finding that the GCA haplotype (rs946053-rs13321-rs2104772) occurred significantly more frequently in the TEN population [27]. Continuing in that direction, they investigated further implications of variants in several genes, including *COL27A1* and *TNC* as well as *IL-6*, *IL-1β* and *CASP8*, concluding that there are subtle effects on protein signaling, interactions or alternate splicing that may be contributing to Achilles tendon pathologies [22].

Further research focused on the whole-exome sequencing approach, where Gibbon et al. sequenced ten healthy controls and ten patients with Achilles tendinopathy by using a platform which included coverage of the untranslated regions as well as miRBase miRNA genes. The results showed four variants in *TNC* (rs1061494, rs1138545, rs2104772 and rs1061495) and three variants in the upstream *COL27A1* gene (rs2567706, rs2241671 and rs2567705) which were genotyped in both Achilles tendinopathy group and anterior cruciate ligament group. The *TNC* gene inferred haplotype was also associated with the risk of Achilles tendinopathy [28].

The inelastic structure of tendons allows resistance to very high forces. The capacity to withstand heavy loads before failure depends on the cross-sectional area and length, but excessive loading and tensile strains will in the end often result in tendinopathies. Extrinsic factors include overuse linked to sports activities, errors in performing exercises, faulty equipment and even weather conditions, for example, sport activities performed in cold weather are considered one of the risk factors [43]. The use of some medications, such as Fluroquinolone based antibiotics, has been proven to impact tendinopathies [44]. On the other hand, intrinsic factors should be taken into consideration for several pathological conditions, for example, the association between Achilles tendinopathy and obesity/weight, genetic aspects as well as age-, gender- and height-related factors of tendinopathy [45].

Professional athletes will have a more significant risk of developing tendinopathies mainly due to overuse from being involved in high-performance sports with the additional negative effect for those being exposed to a wide range of temperatures in sports that take place outdoors [46]. Tendinopathy is increasing in prevalence in professional athletes, as well as in recreational athletes, accounting for substantial part of all sports injuries. Mostly affected tendons are the Achilles and patellar tendons, rotator cuff and extensor carpi radialis brevis, commonly known as the tennis elbow tendon [47,48].

While tendinopathies can often be effectively managed, excessive overuse can still result in tendon rupture. Return-to-sport rates after Achilles tendon repair in NBA players have been reported to be as low as 61%. Up to 30% of NBA players who suffer an Achilles tendon rupture never return to the game. Athletes performing similar movements, including sudden stops, fast changes in direction and explosive acceleration, are all putting increased stress on lower body tendon complexes. Once an Achilles tendon injury occurs, most of the athletes will return to competitive sports, but their careers will be shorter, and their performance will be decreased compared to their previous baseline [49,50]. In 2015. Goodlin et al. performed an interesting pilot program on 14 triathletes, where they were genotyped and educated about their genetic make-up and personal risk profile. Participants responded positively and found the program informative, and it was reported that most of them acted upon their genetic results [51].

## 5. Conclusions

For professional as well as recreational athletes, knowledge of their genetic risk factors could prove to be useful as it contributes to risk of soft tissue injuries. Additional knowledge of risk status could be used in modifying extrinsic factors and taking pre-emptive actions through more thorough conditional training by incorporating more resting periods paired with preventive exercises to reduce the risk of injury. Knowledge of the genetics of sports injuries is still very limited, mainly due to the size of the cohorts that are being investigated, so every piece of additional genetic results for different populations adds to the bigger picture.

## Figures and Tables

**Table 1:** List of primers used for PCR amplification of *COL5A1* rs12722, *COL27A1* rs946053 and *TNC* rs2104772 T>A.

Primer Name	Sequence	5′ Modification	Tm/°C
**COL5A1-F**	5′-GAT TCT GGG TTG CAG TAC CG-3′		60
**COL5A1-R**	5′-AAA GGG GCA CTG GTA CCT G-3′	Biotin	59
**COL27A1-F**	5′-TCC GCT TAC ACC TTC CTT GTA GT-3′		63
**COL27A1-R**	5′-GAA AGG CAC AGG AAG CAC TC-3′	Biotin	60
**TNC-F**	5′-AGC CAC TGG AAA TAA CCC TAC TTG-3′	Biotin	64
**TNC-R**	5′-TTC GTA TTC AGT AGC CTC TCT GAG-3′		64

**Table 2:** List of sequencing primers for pyrosequencing of *COL5A1* rs12722, *COL27A1* rs946053 and *TNC* rs2104772 T>A.

Sequencing Primer Name	Sequence	Tm/°C
**COL5A1-seq**	5′-TAG GAA GTC TCC CCA C-3′	51
**COL27A1-seq**	5′-TGA GCC CCT GCC ACG-3′	54
**TNC-seq**	5′-GCC TCT CTG AGA CCT GT-3′	55

**Table 3:** General characteristics of the tendinopathy group and the control group.

	TEN (n = 63)	Controls (n = 92)	*p*-Value
Age (years)	32.1 ± 12.8	39.0 ± 11.4	**0.006**
Height (cm)	180.5 ± 8.8	179.8 ± 9.6	0.6454
Weight (kg)	79.4 ± 14.9	84.6 ± 15.4	**0.0381**
BMI (kg/m^2^)	24.1 ± 3.6	26.0 ± 3.3	**0.009**
Ethnicity (Caucasian)	100% (63)	100% (92)	1.0000
gender (% male)	75% (47)	78% (92)	0.6639

Ethnicity and gender are represented as a percentage; the remaining variables are expressed as a mean ± standard deviation. Significant *p*-values are in bold. Age, height and weight are self-reported values at the time of the recruitment.

**Table 4:** Allele and genotype frequency.

***COL5A1***	**Allele Frequency**							
SNP	C>T	TEN	CON					
rs12722		n = 63	n = 92	*p*	FDR	OR	95% CI	Association
1	C	42.1% (53)	45.1% (83)	0.5957		0.8835	0.5590–1.3962	none
2	T	57.9% (73)	54.9% (101)	0.5957		1.1319	0.7162–1.7888	none
**Genotype frequency**								
SNP	C>T	TEN	CON					
rs12722		n = 63	n = 92	*p*	FDR	OR	95% CI	Association
11	CC	14.3% (9)	21.7% (20)	0.2456		0.6000	0.2533–1.4210	none
12	CT	55.6% (35)	46.7% (43)	0.2816		14.244	0.7481–2.7121	none
22	TT	30.1% (19)	31.6% (29)	0.8570		0.9381	0.4682–1.8795	none
HWE			0.267					
***COL27A1***	**Allele frequency**							
SNP	G>T	TEN	CON					
rs946053		n = 63	n = 92	*p*	FDR	OR	95% CI	Association
1	G	43.7% (55)	56.5% (104)	**0.0264**	0.0264	0.5959	0.3773–0.9412	**protection**
2	T	56.3% (71)	43.5% (80)	**0.0264**	0.0264	1.6782	1.0625–2.6506	**predisposition**
**Genotype frequency**								
SNP	G>T	TEN	CON					
rs946053		n = 63	n = 92	*p*	FDR	OR	95% CI	Association
11	GG	14.3% (9)	32.6% (30)	**0.0118**	0.0354	0.3444	0.1503–0.7895	**protection**
12	GT	58.7% (37)	47.8% (44)	0.1829		15.524	0.8127–2.9656	none
22	TT	27% (17)	19.6% (18)	0.279		15.193	0.7118–3.2428	none
HWE			0.124					
***TNC***	**Allele frequency**							
SNP	T>A	TEN	CON					
rs2104772		n = 63	n = 92	*p*	FDR	OR	95% CI	Association
1	T	61.1% (77)	48.4% (89)	**0.0276**	0.0276	1.6774	1.0586–2.6579	**predisposition**
2	A	38.9% (49)	51.6% (95)	**0.0276**	0.0276	0.5962	0.3762–0.9447	**protection**
**Genotype frequency**								
SNP	T>A	TEN	CON					
rs2104772		n = 63	n = 92	*p*	FDR	OR	95% CI	Association
11	TT	42.9% (27)	22.8% (21)	**0.0089**	0.0267	25.357	1.2628–5.0918	**predisposition**
12	TA	36.5% (23)	51.1% (47)	0.0745		0.5505	0.2857–1.0608	none
22	AA	20.6% (13)	26.1% (24)	0.4351		0.7367	0.3402–1.5869	none
HWE			0.066					

Allele and genotype frequencies are expressed as percentage with the number of individuals (n) in the parentheses. Results that have been classified as significant by the *p*-value (*p* < 0.05) are in bold and have been additionally checked for false discovery rate (FDR) using the Benjamini–Hochberg adjusted *p*-value. OR—odds ratio. 95% CI—95% confidence interval.

**Table 5:** Haplotype frequency.

Hap Code	Haplotype	CON	TEN	*p*	FDR	OR	95% CI	Association
1	G-A-T	16	5	0.1038		0.4339	0.1547–1.2166	none
2	**G-A-C**	25	8	**0.0424**	**0.0424**	0.4310	0.1880–0.9900	**protection**
3	G-T-T	32	21	0.8678		0.9500	0.5193–1.7380	none
4	G-T-C	31	21	0.9666		0.9871	0.5379–1.8114	none
5	T-A-T	42	30	0.7729		1.0565	0.6186–1.8046	none
6	T-A-C	11	6	0.6441		0.7864	0.2831–2.1843	none
7	**T-T-T**	11	17	**0.0234**	**0.0424**	2.453	1.107–5.434	**predisposition**
8	T-T-C	16	18	0.1219		1.7500	0.8556–3.5792	none

Results that have been classified as significant by the *p*-value (*p* < 0.05) are in bold and have been additionally checked for false discovery rate (FDR) using the Benjamini–Hochberg adjusted *p*-value. OR—odds ratio. 95% CI—95% confidence interval.

## Data Availability

The data presented in this study is available upon request to the corresponding author. Data was not made publicly available to maintain patient privacy.

## Abbreviations

The following abbreviations are used in this manuscript:

COL	collagen
SNP	single-nucleotide polymorphisms
TNC	tenascin C
TEN	tendinopathy
DNA	deoxyribonucleic acid
PCR	polymerase chain reaction
FDR	false discovery rate
CON	control group
BMI	body mass index
HWE	Hardy–Weinberg equilibrium
MMP3	matrix metaloproteinase-3
ESRRB	estrogen-related receptor beta
MiRNA	micro ribonucleic acid
